# From microbiome profiling to clinical translation: designing microbiome studies for clinical actionability

**DOI:** 10.1093/femsle/fnag130

**Published:** 2026-10-22

**Authors:** Natalia G Bednarska

**Affiliations:** Department of Tropical and Infectious Diseases, London School of Hygiene and Tropical Medicine, Faculty of Tropical and Infectious Diseases, London, WC1E 5HT, United Kingdom

**Keywords:** translational, biomarkers, microbiome, type 2 diabetes mellitus, metabolic markers of disease, actionability-focused framework

## Abstract

Microbiome research has identified many highly valuable associations between microbial communities and factors such as diet, antibiotics use, lifestyle, inflammation, infection, and chronic disease. However, many findings remain difficult to translate into clinical practice because they are only association-based and vulnerable to confounding. Many studies also often lack reproducibility. Comparisons between “dysbiotic” and “healthy” microbiome states can be misleading because microbiome profiles are shaped by factors such as diet, medication, geography, host genetics, demographics, disease severity, and reverse causation. Moreover, there remains no universally accepted definition or consensus regarding what constitutes a ‘’healthy’’ microbiota. What in one individual may be considered a healthy mix of species may not necessarily serve another individual whose diet and lifestyle is completely different. The microbiome research must move beyond descriptive profiling toward clinically actionable and mechanistically informed study designs. The key barriers include small cohort sizes, inconsistent sample collection and treatment, differences in sequencing methods, constantly evolving bioinformatic pipelines, limited availability of raw data, and differences between OTU- and ASV-based analyses. To support a large-scale population studies and meta-analysis a transparent reporting along with shared analytical workflows are essential for progress in this field. Microbiome signatures should not only be reproducible, biologically plausible, and modifiable but also linked to the treatment decisions or improved outcomes. This requires longitudinal modelling, external validation, mechanistic studies, and eventually interventional or pragmatic trials. The need for standardization and reproducibility in microbiome research is widely recognized. This paper builds on that foundation by proposing an actionability-focused framework for designing translational microbiome studies. Using Type 2 Diabetes treatment as a worked example demonstrates that microbiome features should be evaluated not only for their statistical associations, but also for the additional predictive value they provide beyond established clinical variables.

## Teaser

This paper proposes a reverse translational, clinical-decision-first framework for microbiome biomarker discovery that integrates standardization, harmonization, Bayesian causal modelling, and early stakeholder engagement to develop clinically actionable biomarkers. ChatGPT (OpenAI) was used to assist with the graphical design and visual refinement of the illustration. The scientific content was developed and verified by the author.

## Introduction

Evidence indicates specific microbiome responses to diet, antibiotics, lifestyle, inflammation, and infections, however so far most of the studies are locked within the association’s patterns without a major shift towards causation or prevention mechanisms (Hong et al. 2024, Walter 2024, Fackelmann et al. 2025). Despite thousands of published association studies, relatively few microbiome discoveries have translated into clinically actionable interventions or reproducible biomarkers. The field has already shifted towards mechanistically informed interventions but still many areas dwell on general “dysbiosis’’ versus “healthy’’ comparisons, which may completely differ because of confounding factors, genetic differences, geographical and demographic factors. Association does not imply causation. Therefore, to establish the relevance of the microbiome to disease, it is important to determine whether and how/if specific microbial changes contribute to disease development. Most microbiome studies identify statistical associations between microbial communities and disease states, providing valuable initial insights into potential microbiome–disease relationships. However, these associations do not establish whether microbial changes contribute to disease development, result from the disease process, or simply reflect external confounding factors.

For example, it was observed by various observational cohorts that pathobionts such as *Klebsiella pneumoniae*, adherent-invasive *Escherichia coli* (AIEC), *Fusobacterium nucleatum, Ruminococcus gnavus* are all associated with Inflammatory Bowel Disease (IBD) dysbiotic ecosystem (Olamafar et al. 2025). However, attempts to broadly suppress these organisms have generally failed to produce consistent or durable clinical remission in humans (Oka and Sartor 2020). These observations suggest that IBD cannot be explained by the presence of individual bacterial taxa alone, but it rather arises from complex interactions among microbial communities, host immune responses, genetic susceptibility, and/or environmental factors (Oka and Sartor 2020). Antibiotic treatment for microbiome modulation is limited by its lack of long-term sustainability and broad-spectrum activity, which can cause non-specific and potentially undesirable alterations to the microbial community and render some bacteria resistant (Cusumano et al. 2025). Consequently, complementary interventions such as prebiotics, probiotics, dietary modifications, and lifestyle changes may be integrated into IBD management to promote sustained microbiome alterations and improve long-term clinical outcomes (Hijová 2025). Importantly, the failure of broad-spectrum bacterial suppression to cure IBD does not imply that these bacteria are unimportant in disease pathogenesis (Batta Gori 1989). Identifying factors that are consistently associated with disease does not necessarily mean removing one factor will reverse an established pathological network. Rather than viewing the failure to identify reliable microbiome biomarkers for IBD as a setback, we should treat it as a lesson for how future biomarker development should proceed.

IBD and Type 2 Diabetes are examples of how microbiome research can become more clinically relevant. Rather than only asking whether the microbiome differs between people with and without diabetes; studies should test whether microbiome features improve prediction of treatment response beyond established clinical variables (such as age, BMI, baseline HbA1c, diabetes duration, medication use, diet, and adherence). Longitudinal cohorts with repeated microbiome, metabolic, inflammatory, and clinical measurements can assess whether baseline or early-treatment microbial features predict changes in HbA1c, fasting glucose, continuous glucose monitoring (CGM) metrics, weight, and medication requirements. Repeated longitudinal sampling is likely to yield more informative biomarkers than cross-sectional studies (Zhou C et al. 2020). Furthermore, prioritizing mechanistically relevant biology over purely associative findings may improve biomarker selection and facilitate faster translation into clinical practice.

The aim of this review is to propose practical frameworks for designing, conducting, and analysing microbiome studies with the central goal of accelerating their clinical translation. Unlike previous reviews, this article provides a comprehensive translational perspective by combining recommendations on study design, early stakeholder engagement, and strategies for overcoming barriers in microbiome biomarker development. It highlights approaches to avoid major study-design limitations and discusses solutions to facilitate the clinical implementation of microbiome-based biomarkers.

## Why association-based microbiome findings often fail to translate

One of the most significant obstacles to interpreting microbiome association studies is the presence of numerous confounding variables that strongly influence microbial composition (Basic et al. 2022). Factors such as diet, medication use, age, lifestyle, ethnicity, geography, socioeconomic status, environmental exposures—can all substantially alter microbial communities independently of disease status (Falony et al. 2016, Rothschild et al. 2018). Diet is particularly influential. Dietary components can rapidly modify gut microbial composition within days, often producing effects larger than those associated with disease states (David et al. 2013). Medication use represents another important confounder. Antibiotics can dramatically disrupt microbial communities, while non-antibiotic drugs such as proton pump inhibitors, metformin, statins, or antidepressants have also been shown to alter microbiome composition (Vich Vila et al. 2020). Geographical variation further complicates interpretation (Suzuki and Worobey 2014). Population-level studies have demonstrated substantial differences in microbiome composition across countries/regions, majorly driven by variations in diet, sanitation, culture, or environmental exposures (Yatsunenko et al. 2012). Table 1 highlights limited metadata as a key barrier to microbiome translation. This could be improved by including not only clinical, dietary, and medication-related metadata, but also lifestyle and treatment factors such as physical activity and lifestyle changes. Where feasible, additional potential confounders, such as socioeconomic status, could also be captured. Even when not all variables can be collected across studies, harmonized terminology and standardized levels would improve comparability.

**Table 1:** Barriers to microbiome biomarker translation and proposed solutions

Barrier	Consequence	Proposed solution
Small cohorts	Unstable associations	Larger longitudinal cohorts and external validation
Heterogeneous sequencing	Poor comparability	Standardized protocols and shared pipelines
Limited metadata	Residual confounding	Harmonized clinical, dietary, medication, and lifestyle metadata; where feasible, inclusion of potential confounders (e.g. socioeconomic status) using standardized terminology and levels.
OTU/ASV inconsistency	Difficult meta-analysis	Raw data deposition and common reprocessing
Cross-sectional design	Weak causal inference	Longitudinal modelling and mechanistic validation
Poor clinical framing	Limited implementation	Define clinical decision before biomarker discovery

Multiple global microbial data sets are conducted using different sequencing primers, dissimilar sequencing platforms and many times the raw data is not deposited for re-analyses. Moreover, there is still changing bioinformatic environment for data analysis pipelines. Different microbiome studies have also used different approaches to defining microbial features, particularly operational taxonomic units (OTUs) and amplicon sequence variants (ASVs). OTUs group similar sequences together, often using a 97% similarity threshold, and were widely used in earlier 16S rRNA studies. By contrast, ASVs infer exact biological sequences after sequencing error correction, claiming to provide a higher-resolution and more reproducible sequence features across studies. In contrast to conventional Operational Taxonomic Unit (OTU)-based methods, ASVs detect sequence variations at a single-nucleotide level, enabling enhanced precision and reliability. However, whether this increased resolution consistently improves downstream biological interpretation or clinical prediction remains an area of ongoing evaluation (Callahan et al. 2017). This matters for large population microbiome research because results generated using OTU-based pipelines are not directly comparable with results generated using ASV-based pipelines (Sinha et al. 2017, Jeske and Gallert 2022). Therefore, when moving from small cohorts to large multi-site studies, researchers need to harmonize bioinformatic workflows, clearly report whether OTUs or ASVs were used, preserve raw sequence data where possible, and, ideally, reprocess datasets through a common pipeline. This improves comparability, reproducibility, and the ability to validate microbiome signatures across populations. If all methodology would be aligned, we would get much more information from small association studies, which data would also be beneficial for Bayesian and inferential modelling.

The fragmentation of microbiome research methodology makes combined efforts to tackle the answers through metanalysis impossible. Ideally common standardization pipelines and data preparation workflows need to be harmonized and be available for everyone wanting to publish their microbiome data. The human microbial system is so complex with numerous confounders, which could be simply taken into larger similar cohort to draw better statistical conclusions. In 2025 the Human Microbiome Action consortium initiated a Delphi survey to establish a consensus on the needs, challenges, and limitations in developing qualified microbiome-based biomarkers. Around 307 experts participated in this survey, sadly as much as 78% of experts were not aware of biomarker definitions and qualification procedures (Rodriguez et al. 2025).The paucity of validated analytical methods appears to be the principal factor hindering the qualification of these biomarkers. The survey also showed that clinical implementation of these biomarkers would only be possible if standardized and validated molecular assay kits with simple interpretation were developed (Rodriguez et al. 2025). This initiative serves as a foundation for designing and implementing public-private collaborative projects to overcome the challenges and promote clinical application of microbiome-based biomarkers.

In the Type 2 Diabetes example, the question is not only whether the microbiome differs between people with and without diabetes, but also whether microbiome profiles can improve the prediction of future T2D development or treatment response beyond established clinical factors (such as age, BMI, baseline HbA1c, diabetes duration, medication use, diet, and treatment adherence). Identifying microbiome profiles associated with increased T2D risk could support earlier intervention, including targeted lifestyle and dietary changes and, where appropriate, metformin therapy. This approach would make microbiome findings clinically relevant rather than simply descriptive.

Many microbiome studies employ cross-sectional designs in which microbial samples are collected after disease onset. Under these circumstances, it is often impossible to determine whether microbiome alterations contribute to disease development or arise because of disease-related physiological changes. In inflammatory bowel disease, for example, intestinal inflammation changes oxygen availability, nutrient composition, immune activity, and epithelial integrity, all of which influence microbial ecology (Lloyd-Price et al. 2019). Consequently, observed microbial alterations may represent downstream effects of inflammation rather than causal drivers. Longitudinal cohort studies, Mendelian randomization approaches along with mechanistic experiments (e.g. using *in vitro* experiments or germ-free animal models) can help addressing reverse causation.

## Designing microbiome studies for clinical actionability

A proposed solution to improve clinical actionability is to strengthen causal inference frameworks in the design and planning of preclinical and clinical microbiome studies (Khelfaoui et al. 2026).

Causal inference frameworks are approaches used to determine whether an observed relationship between two factors reflects a causal effect rather than a simple association, while accounting for potential confounding factors (Lv et al. 2021). Microbiome-based interventions should therefore be strategically designed around clearly defined hypotheses, with methods and outcome measures sufficiently standardized to enable direct comparison across studies (examples of such measures described in Table 2). This would facilitate subsequent meta-analytic research by increasing the comparability of findings across different study populations and settings. Causal inference frameworks could then be applied across these studies, strengthening the overall evidence base by enabling causal effects to be assessed across larger and more diverse pooled samples. The efforts to standardize reporting microbiome research were made in the past decade, for example through a design of Strengthening The Organization and Reporting of Microbiome Studies (STORMS reporting checklist) (Mirzayi et al. 2021). The checklist was developed by a large group of stakeholders comprising of microbiologists, epidemiologists, biostatisticians, bioinformaticians and physician-scientists to address the challenges of reporting microbiome studies. STORMS consists of a 17-item checklist covering key aspects of study design, participant characteristics, laboratory methods, bioinformatics, statistical analysis and reporting (Mirzayi et al. 2021). However, the STORMS guidelines were designed primarily to support researchers in producing and assessing complete and reproducible microbiome studies, rather than to prescribe specific experimental or analytical methods. STORMS has been promoted to journals and researchers, but its use is not universally mandatory. Therefore, to date microbiome research remains highly fragmented and heterogeneous, with substantial variation in study designs, methodologies, and outcome measures. This limits direct comparison between studies and currently constrains the application of causal inference frameworks across the wider evidence base. It would be highly advisable to incorporate STORMS principles from the earliest stages of study design through to publication to promote more reproducible and comparable human microbiome research globally. Journals could also require STORMS as part of the publication process, like PRISMA, which is widely required for systematic reviews.

**Table 2:** Example actionability endpoints in Type 2 diabetes microbiome research

Domain	Example measure	Clinical relevance
Clinical response	HbA1c reduction ≥0.5% or ≥1.0%*	Treatment effectiveness
Metabolic response	Fasting glucose, CGM time-in-range	Glycaemic control
Microbiome function	SCFA, bile acid, BCAA pathways	Mechanistic plausibility
Treatment prediction	Response to metformin, GLP-1RA, SGLT2i	Precision treatment selection
Implementation	Validated assay, interpretable report	Clinical usability

* please note that the percentage measure is set as exemplary and may not reflect clinical significance

One of the most efficient designs in clinical microbiome studies is building longitudinal, fully phenotyped cohort with well specified framework from association through mechanisms and intervention. Precision microbiome medicine should ideally integrate host phenotype with microbiome functional activity and the bidirectional microbial–host metabolic pathways through which the microbiome influences host physiology and, in turn, host physiology influences the microbiome (Tremaroli and Bäckhed 2012, Vich Vila et al. 2020). Ideally, patient classification should also incorporate treatment context rather than relying solely on a healthy/unhealthy categorization, as this binary distinction may be overly simplistic and ambiguous. Treatment context should account for factors such as medication use, treatment intensity, lifestyle interventions, or concomitant therapies—which may independently influence both the host phenotype and the microbiome. Because of the contrasting data findings across the world’s commercial microbiome tests remain unreliable for individual clinical decisions. If I were to design my next microbiome study, I would aim to identify modifiable microbial functions that predict clinically meaningful outcomes that can be further tested in precision interventions. A single baseline microbiome sample may show associations, but repeated sampling can show whether microbiome features change after treatment and whether early microbiome shifts predict later improvement in HbA1c, fasting glucose, CGM time-in-range, weight, inflammatory markers, or medication requirements. This approach would be especially relevant for comparing treatments strategies such as metformin, GLP-1 receptor agonists, SGLT2 inhibitors, dietary interventions, exercise interventions, or combination therapy. Specifically, the primary endpoint would be improved prediction of treatment response using microbiome parameters alongside established clinical factors. This could help in distinguishing responders from non-responders and in supporting more precise treatment selection. For example: the primary endpoint could be the incremental predictive value of baseline and early-treatment microbiome species or functional profiles for achieving a ≥0.5% or ≥1.0% reduction in HbA1c at 6 months, beyond established clinical factors including age, BMI, baseline HbA1c, diabetes duration, medication class, diet, or adherence (see more examples in Table 2). Secondary microbiome endpoints could assess shifts in specific taxa, functional pathways and metabolites associated with treatment response. This design would help to identify potential microbiome-mediated mechanisms and therapeutic targets. Therefore, in this example, incremental predictive value refers to the additional improvement in predicting treatment response gained by adding microbiome information to established clinical factors. A practical example of such a study design would be to investigate whether metformin improves HbA1c and whether microbiome features can help to explain or predict this response. The primary clinical endpoint could be the change in HbA1c following metformin treatment. Treatment response can be classified according to changes in HbA1c, providing a basis for distinguishing responders and non-responders. Baseline microbiome composition and function could then be assessed to determine whether specific microbial features improve prediction of treatment response beyond established clinical factors (Kim et al. 2022). Repeated microbiome sampling could further determine whether early changes in specific taxa, such as an increase in *Akkermansia* spp., are consistently associated with subsequent improvement in HbA1c (Wu et al. 2017). Such findings could help to identify microbiome features that may contribute to treatment response and could potentially be targeted in future precision microbiome interventions.

For T2DM microbiome research, strengthening causal inference could involve longitudinal studies comparing different treatment strategies and their effects on the gut microbiome, glycaemic biomarkers, and clinical outcomes. Such designs would enable the identification of microbiome features that predict or mediate treatment response, thereby increasing the translational relevance of microbiome research and supporting precision medicine approaches.

In interventional research on Type 2 Diabetes Mellitus, microbiome actionability endpoints are increasingly defined in terms of predictive value and mechanistic insight (Jing and Jiang 2025). The aim is to move beyond glycaemic outcomes alone. One key endpoint is whether gut microbiome profiles improve prediction of HbA1c response beyond baseline clinical variables. This reflects a shift toward precision stratification of treatment response (Zeevi et al. 2015).

Another endpoint is the use of microbiome signatures to predict response to glucose-lowering therapies (Klemets et al. 2026). This includes metformin, GLP-1 receptor agonists, SGLT2 inhibitors, insulin, dietary interventions, and bariatric surgery. Metformin is the best characterized example. It consistently induces reproducible shifts in gut microbial composition linked to glycaemic effects (Forslund et al. 2015, Bauer et al. 2018). Bariatric surgery also produces major and durable microbiome changes associated with metabolic improvement (Tremaroli and Bäckhed 2012, Liou et al. 2013).

Interventional studies also assess therapy-associated shifts in taxa, species and functional pathways. These changes are often rapid and partially reversible. Attention should be given to microbial functions involved in host metabolism and inflammation. Mechanistic endpoints focus on key microbial metabolic pathways. Short-chain fatty acid production is frequently assessed. These metabolites influence insulin sensitivity and energy balance (Canfora et al. 2015). Bile acid metabolism is also important, as it affects glucose regulation through FXR and TGR5 signalling (Sayin et al. 2013). Branched-chain amino acid metabolism is another pathway linked to insulin resistance (Newgard et al. 2009). Inflammation-related microbial functions are also commonly included. These functions include microbial activities that either directly or non-directly influence intestinal barrier integrity and levels of immune activation. In individuals with type 2 diabetes, alterations in the gut microbial community may increase the abundance or activity of bacteria capable of producing pro-inflammatory compounds, particularly lipopolysaccharide (LPS) (Chong et al. 2025). Increased intestinal permeability may facilitate the translocation of LPS and other microbial products into the circulation, contributing to metabolic endotoxaemia and activation of innate immune pathways, including Toll-like receptor 4 (TLR4) and NF-κB signalling (Chong et al. 2025).This inflammatory response is associated with increased production of pro-inflammatory cytokines, including TNF-α and IL-6, which can interfere with insulin signalling and contribute to insulin resistance, a key feature of type 2 diabetes (Barlow and Mathur 2023).

Finally, metabolite-level endpoints are used to link microbial change to clinical response. These include short-chain fatty acids, bile acid derivatives, and other circulating microbial metabolites. Such measures help clarify whether microbiome shifts are causal mediators of improved glycaemic control (Vrieze et al. 2012).

So why the longitudinal design is optimal here? Because it allows comparison of the treatment groups longitudinally but most importantly allow to model each person as their own baseline control.

### How do we go from small cohorts into large populational studies of microbiome?

To move from small clinical microbiome cohorts to larger population studies, we need: standardized sampling, harmonized metadata (including treatment exposure and other relevant clinical variables), interoperable clinical data, shared analytical pipelines as well as external validation across diverse populations.

Standardization allows us to compare microbiome data while harmonization helps to combine it. Both are essential for moving from small discovery cohorts to population-scale precision microbiome medicine. Small cohorts are useful for deep mechanistic discovery, but population studies require comparable protocols and shared data models so that findings can be replicated across sites and populations. In Type 2 Diabetes microbiome research, this means harmonizing treatment exposure, HbA1c response definitions, medication use, diet, antibiotic exposure, metabolic biomarkers and microbiome functional profiling. Only then can microbiome signatures move from an exploratory association to validated predictors of treatment response along with actionable precision-medicine tools.

Clinical action requires stronger evidence than cross-sectional association. This may include longitudinal modelling, mediation analysis, replication in external cohorts, experimental validation in organoids or animal models, and eventually randomized or pragmatic trials (Capili and Anastasi 2025). The aim is to demonstrate that a microbiome feature is not only correlated with treatment response, but also biologically plausible, reproducible, and useful for guiding care. Ideally, bench and clinical research would be integrated within a larger translational study, combining patient cohorts, multi-omics profiling, mechanistic experiments, and clinical outcome assessment. This approach would provide stronger evidence for biomarker validity and clinical utility, although cost, infrastructure, sample size, and interdisciplinary coordination may be major limitations (Ou et al. 2021). Alternatively, parallel research programmes can also be effective, where clinical cohort studies identify candidate microbiome biomarkers while separate mechanistic studies investigate the biological pathways underlying these associations. Together, these complementary approaches can help move microbiome research from association-based discovery toward clinically actionable precision medicine.

In Type 2 diabetes microbiome research, a taxon, pathway, or metabolite should not be treated as actionable just because it differs between responders and non-responders (Pepe 2003). It must be tested over time to see if it consistently predicts glycaemic response. It optimally should also be validated in independent cohorts. Finally, it needs a plausible mechanism, such as short-chain fatty acid production, bile acid metabolism, inflammation, gut barrier function, or drug–microbiome interactions. The strongest design would integrate clinical follow-up with mechanistic validation, but where this is not feasible, parallel clinical and laboratory studies can still provide complementary evidence.

### Stakeholder engagement for actionable translational research

Another important point when thinking about study design for rapid translational results is early engagement with stakeholders. Actionability should be assessed not only by improved prediction of treatment response, but also by clinical interpretability, patient acceptability, feasibility of implementation. What matters the most is the therapeutic/diagnostic potential to improve outcomes without widening inequalities. For that reason, patients, clinicians, laboratory scientists, bioinformaticians, policymakers, regulators, and industry partners should be involved from the earliest stages of study design. Since policymakers and regulators define implementation requirements it is important to consider them during the project planning process. Looking from the perspective of policymakers would drive the research aims towards precise evidence generation to make a positive breakthrough. Clinicians are also central stakeholders because they help define what “actionable” means in real care. For example, a diabetologist, General Practitioner (GP) diabetes nurse, pharmacist, or dietitian can advise whether a microbiome-based prediction would influence treatment choice, dietary advice, or monitoring. They can also help deciding what level of prediction improvement would justify adding microbiome testing to standard clinical care. In T2DM case this could mean asking whether microbiome profiles improve prediction of HbA1c response, whether they identify likely responders or non-responders to specific treatments, and whether that information could change treatment decisions.

Ethics, governance, and data protection stakeholders are particularly important in microbiome research because microbiome data can reveal information about diet, lifestyle, disease risk, medication exposure, geography, and potentially sensitive health patterns. If microbiome data are linked to electronic health records, glucose monitoring, diet apps, or genomic data, then consent, privacy, data sharing, and return of results need careful planning. Stakeholder engagement can help decide whether individual microbiome results should be returned to participants, how uncertainty should be communicated, and how to avoid overstating clinical meaning.

### Reverse translation from clinical decision to microbiome biomarker

Most of the microbiome studies are exploratory and follow the path from microbial difference to association to a possible list of biomarkers followed by possible intervention study. However, what if we try slightly different approach? What if we start with a study in which methodological design begins with a defined clinical decision rather than a microbial association? Faecal microbiota transplantation (FMT) illustrates a decision-first therapeutic paradigm, in which microbiome knowledge is directly linked to a predefined clinical intervention. This contrasts with the more common exploratory biomarker discovery pipeline and aligns conceptually with decision-centric biomarker development (Baunwall et al. 2020).

In Type 2 Diabetes, this means first asking whether microbiome information would change treatment selection, escalation, monitoring, or dietary intervention, and then designing longitudinal microbiome studies to test whether microbial features provide sufficient incremental predictive value, mechanistic plausibility, and clinical utility to support that decision. For example, rather than asking which microbial taxa are associated with Type 2 Diabetes, a decision-first research question would ask: Among patients whose HbA1c remains above target, does microbiome profiling improve the choice between adding a glucagon-like peptide-1 (GLP-1) receptor agonist versus an sodium-glucose cotransporter-2 (SGLT2) inhibitor?

A decision-first framework shifts the focus from finding statistically significant microbial associations to demonstrating clinical utility. The microbiome only has value if it changes patient management, improves prediction or leads to better patient outcomes. Reverse translation framework is depicted on the diagram 1 (Fig. 1) and further described in the following paragraphs. Panel 1 of the Fig. 1. shows the global collation of association studies across diverse populations, with harmonized metadata and high-throughput microbiome profiling used to identify reproducible association signals. This is followed by application of Bayesian modelling and uncertainty quantification to obtain robust and calibrated effect estimates from these heterogeneous datasets. Panel 3 integrates multiple data types and causal assumptions to move beyond association. Steps 1–3 of this framework has already been attempted in T2D research. There are multi-cohort/meta-analytic studies, and several studies have applied causal approaches such as Mendelian randomization. However, these studies also illustrate the remaining limitations: cohort, population, treatment, sequencing, and analytical heterogeneity can lead to inconsistent microbial signatures. For example, early studies had already reported differences in gut microbial composition in individuals with T2D (Larsen et al. 2010). This was followed by larger, metagenome-wide association studies by Wang et al. (2012), which identified and validated thousands of T2D-associated microbial markers. Their work showed that medication such as metformin, can substantially confound T2D-associated microbiome signatures (Wang et al. 2012). A 2026 multi-cohort metagenomic analysis demonstrates that cross-population integration is feasible, while emphasizing that cohort-specific noise can distort T2D-associated microbial signatures (Dong et al. 2026). These findings confirm that Steps 1–3 shown on Fig. 1 are technically feasible and have already been applied in T2D, but their translation into robust and generalizable causal biomarkers remains limited by heterogeneity and incomplete harmonization across cohorts. Steps 4–6 of Fig. 1 therefore provide the framework for addressing these limitations by translating causal hypotheses into appropriately designed clinical studies (Panel 4), prospectively validating their clinical relevance and reproducibility (Panel 5), and feeding these findings back into the earlier stages to refine associations, causal models and subsequent study designs (Panel 6). This iterative feedback loop has the potential to improve the efficiency and precision of clinical study design, reduce unnecessary or poorly targeted studies, resulting in lower the costs associated with microbiome-based research and development.

**Figure 1:**
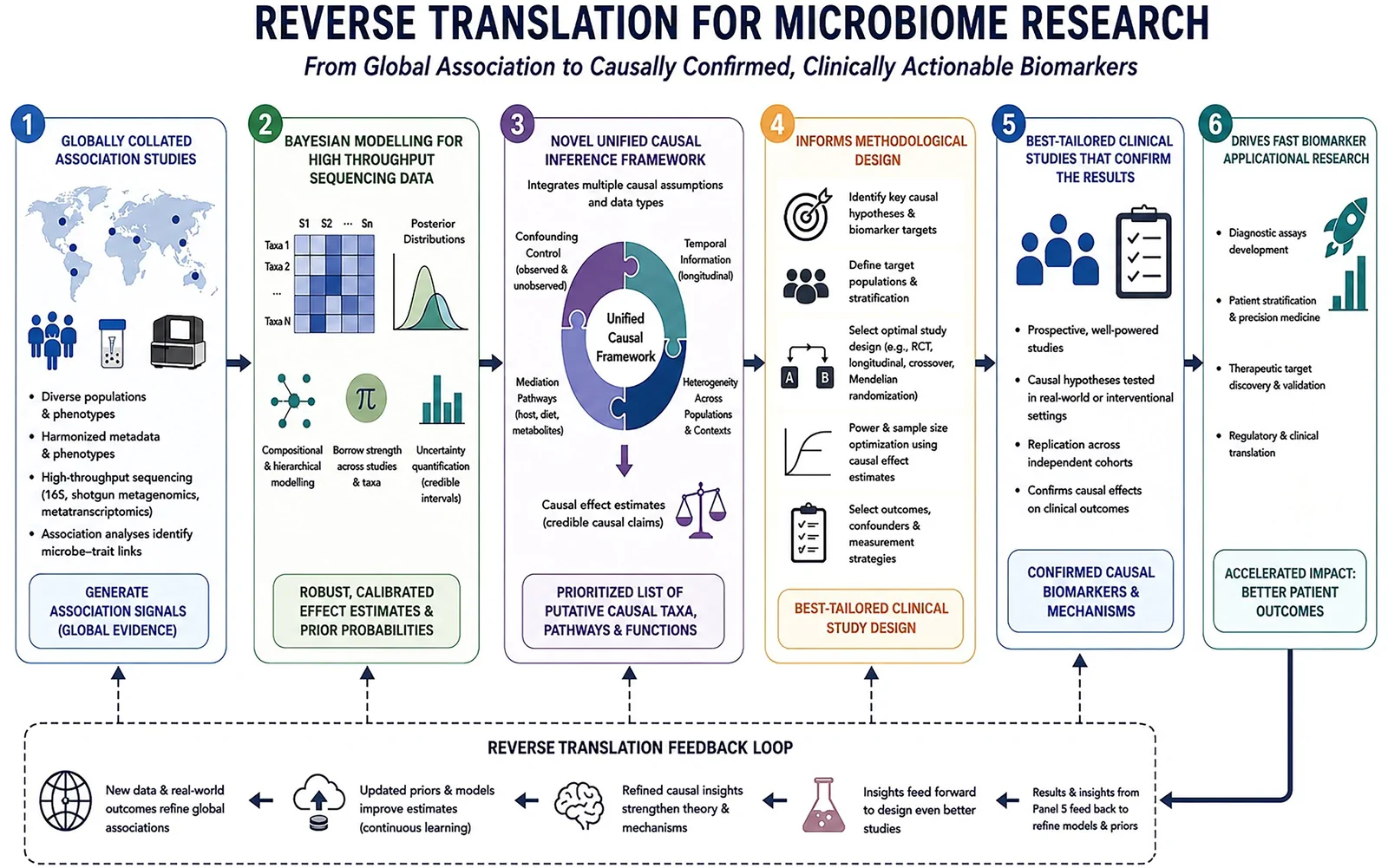
Reverse translation framework for microbiome research. Globally collated microbiome association studies are integrated using Bayesian modelling of high-throughput sequencing data and a unified causal inference framework to identify robust causal relationships. These findings guide optimally tailored clinical study design, enabling targeted validation of causal mechanisms and accelerating the translation of microbiome biomarkers into clinical applications. ChatGPT (OpenAI) was used to assist with the graphical design and visual refinement of the illustration. The scientific content was initially developed and verified by the authors.

### Bayesian modelling for high-throughput sequencing data and using a novel unified causal inference framework for causal inference

In addition to confounding, the statistical properties of gut microbiome data further complicate downstream analyses. Taxonomic abundances are compositional, meaning that each measurement represents a proportion of the total microbial community rather than an absolute count. Consequently, an increase in the relative abundance of one taxon can result in apparent decreases in others, even in the absence of changes in their absolute abundance. This compositional constraint can induce spurious correlations and complicate the use of conventional statistical methods that do not account for the dependence among taxon proportions. A range of methodological approaches has been developed to strengthen causal inference in microbiome research, including directed acyclic graphs (DAGs) to formalize causal assumptions, Mendelian randomization (MR) using host genetic instruments, longitudinal models to establish temporal ordering, double machine learning (DML) for effect estimation in high-dimensional settings, and mediation analysis (to investigate biological mechanisms linking microbial features to host outcomes) (Lv et al. 2021, Jiang and Li 2026).

Mendelian randomization has been increasingly used to investigate potential causal relationships between the gut microbiome and T2D. For example, Ruan et al. (2025) used bidirectional two-sample MR to investigate gut microbiota across four T2D subtypes—SIDD, SIRD, MOD, and MARD (Ruan et al. 2025). The study aimed at identifying subtype-specific associations and potential bidirectional relationships. Sensitivity analyses were performed to assess heterogeneity and horizontal pleiotropy. However, the authors emphasized that further validation is required before these findings can be considered clinically relevant. More recently, Zhou et al. (2025) extended this approach by combining bidirectional MR with two-step mediation MR to investigate potential pathways linking gut microbiota, circulating inflammatory cytokines and T2DM (Zhou X et al. 2025). Using four microbiome GWAS datasets and T2DM data from ∼1.53 million individuals, they identified IFN-γ as a potential mediator of the association between *Kandleria vitulina* and T2DM (Zhou et al. 2025). However, the findings did not survive multiple-testing correction. The findings highlight the need for further experimental and clinical validation. These studies illustrate how MR and mediation approaches can move microbiome research beyond simple association towards testable causal and mechanistic hypotheses. They also demonstrate the obvious limitations of applying individual causal methods in isolation. Building on these advances, a recently published causal inference framework integrates complementary approaches within a unified strategy for evaluating causality (Ascandari et al. 2026).

The suggested framework distinguishes unsupported claims from those supported by evidence by separating assumptions from causal identification and interpretation. The computational strategy designed by Ascandari et al. (2026) aim to solve the translational failure by subjecting multi-cohorts and public metagenomic datasets into the workflow. The unified workflow presented by Ascandari et al. (2026) belongs to an emerging, cutting-edge subfield known as Causal Machine Learning (CausalML) for the microbiome. The CausalML is a powerful online tool with a built-in automated batch correction for massive multi-cohort data consolidation (Koh et al. 2025). The framework proposed by Ascandari et al. focuses primarily on the transition from association to causal interpretation and intervention design. It provides methodological tools and decision points that strengthen the causal-inference stage (Fig. 1, Panel 3) of the framework presented in this publication (Ascandari et al. 2026). These platforms share the same architectural philosophy: shifting the microbiome field away from pure correlation toward reproducible, causal target identification. Other emerging microbial causal machine learning platforms utilize hybrid approaches. For example, Khelfaoui et al., (2026) suggested pairing Double Machine Learning (Double ML) and Deep Instrumental Variables (Deep IV) to map out non-linear, high-dimensional microbial noise and accurately predict the success of microbiome-mediated policy or clinical interventions. Double/debiased machine learning enables estimation of treatment effects in settings with high-dimensional covariates while using flexible machine-learning models for nuisance parameters (Chernozhukov et al. 2018).

Choice of analytical platform depends on specific goals, with MiCML providing the fastest, automated, cloud-based workflow for large-scale, multi-cohort analyses (Koh et al. 2025). For higher accuracy and avoiding false positives in translation, Ascandari’s approach serves best, while Deep Instrumental Variable (Deep IV) methods are optimal for handling complex, non-linear biological data. Please note however that Deep IV itself is not originally a microbiome method. Its application to microbiome research is much newer (Hartford et al. 2017). Bayesian modelling techniques on the other hand are excellent tools in microbiome to reduce the noise and sparsity problem. Moreover, since microbes cooperate, compete and depend on each other, the Bayesian Network (BN) modelling can help to predict how a change in one microbe/taxa (e.g. due to antibiotics) will cascade and affect the rest of the ecosystem (Verster et al. 2022). This has an enormous power in multicausal diseases. Overall, combining global microbiome datasets into harmonized analyses, followed by causal machine learning and Bayesian modelling, could strengthen evidence for microbiome biomarker discovery. These complementary approaches may improve causal inference, reduce uncertainty and help distinguishing true causal relationships from simple associations. However, these approaches will require standardized microbiome data and open, interoperable databases to enable robust and highly reproducible analyses.

## Conclusion

The main aim of this paper was to propose a research framework that integrates methodological rigour with clinical decision-making to accelerate the translation of microbiome discoveries into clinically useful applications. Standardization allows microbiome methods to be consistent whilst harmonization allows data to be compared and combined. Additionally, an early stakeholder engagement ensures that the research addresses real-world needs. Altogether, these three elements help microbiome studies move from small exploratory cohorts to large population studies and eventually to precision microbiome medicine. Methodological standardization, including the adoption of reproducible sequence-variant approaches such as amplicon sequence variants (ASVs) rather than operational taxonomic units (OTUs), is important for improving comparability across microbiome studies (Callahan et al. 2017). Transparent reporting and harmonization are equally important for comparing findings across cohorts, as emphasized by the STORMS guidelines for human microbiome research (Mirzayi et al. 2021). Using treatment response in Type 2 Diabetes as an example, it becomes clear that microbiome features should be evaluated on more than statistical association alone. To be clinically useful, studies should demonstrate incremental predictive value, biological plausibility, and the potential for therapeutic modification. Microbiome-based markers must also be clinically interpretable, enabling treatment decisions that can directly improve patient outcomes. Achieving this requires a shift toward a clinical-decision-first framework, an approach that some recent studies have already begun to adopt. This represents the next step in translating microbiome research from descriptive knowledge into practical, clinically applicable tools. Clinical data should be collected within a Bayesian modelling framework that explicitly incorporates potential confounding and, where appropriate, multicausal relationships. Bayesian methods allow prior information to be formally combined with newly collected data, enabling posterior estimates to be updated as additional evidence becomes available (Goligher et al. 2024). Clinical data should be collected within a robust strategic Bayesian modelling framework that accounts for potential confounders and incorporates multicausal models. This approach allows newly collected clinical data to iteratively inform and update the model as the research progresses. In turn, the evolving model provides evidence-based decision points that can guide study conduct, optimize data collection, and adapt the research design to maximize the strength and validity of causal inference

Early stakeholder engagement (such as patients, clinicians, laboratory scientists, bioinformaticians, policymakers, regulators, industry partners) is another essential factor to ensure feasibility and equitable implementation of the microbiome research.

## References

[bib1] Ascandari AA, Aminu S, Benhida R et al. From association to causation: a decision-aware framework for reproducible biomarker discovery and precision intervention design in the human gut microbiome. Brief Bioinform. 2026;27:bbag316. 10.1093/BIB/BBAG316 PMC13271389 42302279

[bib2] Barlow GM, Mathur R. Type 2 diabetes and the microbiome. J Endocr Soc. 2023;7:bvac184. 10.1210/JENDSO/BVAC184 PMC9777768 36578879

[bib3] Basic M, Dardevet D, Abuja PM et al. Approaches to discern if microbiome associations reflect causation in metabolic and immune disorders. Gut Microbes. 2022;14:2107386. 10.1080/19490976.2022.2107386. PMC9361767 35939623

[bib4] Batta Gori G. Epidemiology and the concept of causation in multifactorial diseases. Regul Toxicol Pharm. 1989;9:263–72. 10.1016/0273-2300(89)90065-2. 2756173

[bib5] Bauer PV, Duca FA, Waise TMZ et al. Metformin alters upper small intestinal microbiota that impact a glucose-SGLT1-sensing glucoregulatory pathway. Cell Metabolism. 2018;27:101–117.e5. 10.1016/j.cmet.2017.09.019. 29056513

[bib6] Baunwall SMD, Lee MM, Eriksen MK et al. Faecal microbiota transplantation for recurrent Clostridioides difficile infection: an updated systematic review and meta-analysis. EClinicalMedicine. 2020;29-30:100642. 10.1016/j.eclinm.2020.100642. PMC7788438 33437951

[bib7] Callahan BJ, McMurdie PJ, Holmes SP. Exact sequence variants should replace operational taxonomic units in marker-gene data analysis. ISME J. 2017;11:2639–43. 10.1038/ismej.2017.119. PMC5702726 28731476

[bib8] Canfora EE, Jocken JW, Blaak EE. Short-chain fatty acids in control of body weight and insulin sensitivity. Nat Rev Endocrinol. 2015;11:577–91. 10.1038/nrendo.2015.128. 26260141

[bib9] Capili B, Anastasi JK. Pragmatic clinical trials: a study design for real-world evidence. Am J Nurs. 2025;125:56–58. 10.1097/AJN.0000000000000014. PMC11902901 39844235

[bib10] Chernozhukov V, Chetverikov D, Demirer M et al. Double/debiased machine learning for treatment and structural parameters. Econom J. 2018;21:C1–C68. 10.1111/ECTJ.12097.

[bib11] Chong S, Lin M, Chong D et al. A systematic review on gut microbiota in type 2 diabetes mellitus. Front Endocrinol. 2025;15:1486793. 10.3389/FENDO.2024.1486793 PMC11782031 39897957

[bib12] Cusumano G, Flores GA, Venanzoni R et al. The impact of antibiotic therapy on intestinal microbiota: dysbiosis, antibiotic resistance, and restoration strategies. Antibiotics. 2025;14:371. 10.3390/ANTIBIOTICS14040371. PMC12024230 40298495

[bib13] David LA, Maurice CF, Carmody RN et al. Diet rapidly and reproducibly alters the human gut microbiome. Nature. 2013;505:559–63. 10.1038/nature12820. PMC3957428 24336217

[bib14] Dong Y, Wang M, Zhou X et al. Multi-cohort analysis of metagenome for type 2 diabetes identified universal gut microbiota signatures across populations. Nutr Diabetes. 2026;16:9. 10.1038/s41387-026-00418-w. PMC13057204 41927536

[bib15] Fackelmann G, Manghi P, Carlino N et al. Gut microbiome signatures of vegan, vegetarian and omnivore diets and associated health outcomes across 21,561 individuals. Nat Microbiol. 2025;10:41–52. 10.1038/s41564-024-01870-z. PMC11726441 39762435

[bib16] Falony G, Joossens M, Vieira-Silva S et al. Population-level analysis of gut microbiome variation. Science (1979). 2016;352:560–4. 10.1126/SCIENCE.AAD3503;REQUESTEDJOURNAL:JOURNAL:SCIENCE;CTYPE:STRING:JOURNAL. 27126039

[bib17] Forslund K, Hildebrand F, Nielsen T et al. Disentangling type 2 diabetes and metformin treatment signatures in the human gut microbiota. Nature. 2015;528:262–6. 10.1038/nature15766. PMC4681099 26633628

[bib18] Goligher EC, Heath A, Harhay MO. Bayesian statistics for clinical research. The Lancet. 2024;404:1067–76. 10.1016/S0140-6736(24)01295-9. PMC12051211 39277290

[bib19] Hartford J, Lewis G, Leyton-Brown K et al. Deep IV: a flexible approach for counterfactual prediction. In: Proceedings of the 34th International Conference on Machine Learning. PMLR, 2017, 1414–23. https://proceedings.mlr.press/v70/hartford17a.html (13 Oct. 2026, date last accessed).

[bib20] Hijová E. Probiotics in IBD: evidence and perspectives on patient health and disease management. IJMS. 2025;26:9065. 10.3390/IJMS26189065. PMC12469838 41009629

[bib21] Hong Y, Li H, Chen L et al. Short-term exposure to antibiotics begets long-term disturbance in gut microbial metabolism and molecular ecological networks. Microbiome. 2024;12:80. 10.1186/S40168-024-01795-Z. PMC11075301 38715137

[bib22] Jeske JT, Gallert C. Microbiome analysis via OTU and ASV-based pipelines—a comparative interpretation of ecological data in WWTP systems. Bioeng. 2022;9:146. 10.3390/BIOENGINEERING9040146. PMC9029325 35447706

[bib23] Jiang Z, Li G. Microbiome mediation analysis: methods, assumptions, and practical considerations. Front Cell Infect Microbiol. 2026;16:1832981. 10.3389/FCIMB.2026.1832981 PMC13191630 42180244

[bib24] Jing M, Jiang Y. Microbiome−mediated crosstalk between T2DM and MASLD: a translational review focused on function. Front Endocrinol (Lausanne). 2025;16:1677175. 10.3389/FENDO.2025.1677175/FULL. PMC12665569 41334445

[bib25] Khelfaoui I, Wang W, Shehata AI et al. STROBE-causal machine learning for the human microbiome: systematic review on methodological innovations and validation frameworks. Front Microbiol. 2026;17:1705116. 10.3389/FMICB.2026.1705116/FULL. PMC13057537 41960430

[bib26] Kim H, Jung DY, Lee SH et al. Long-term changes in HbA1c according to blood glucose control status during the first 3 months after visiting a tertiary university hospital. J Korean Med Sci. 2022;37:e281. 10.3346/JKMS.2022.37.E281 PMC9530310 36193638

[bib27] Klemets A, Reppo I, Krigul KL et al. Fecal microbiome predicts treatment response after the initiation of semaglutide or empagliflozin uptake. Sci Rep. 2026;16:6126. 10.1038/s41598-026-36318-3. PMC12902008 41580554

[bib28] Koh H, Kim J, Jang H. MiCML: a causal machine learning cloud platform for the analysis of treatment effects using microbiome profiles. BioData Mining. 2025;18:10. 10.1186/S13040-025-00422-3. PMC11783787 39885552

[bib29] Larsen N, Vogensen FK, Van Den Berg FWJ et al. Gut microbiota in human adults with type 2 diabetes differs from non-diabetic adults. PLoS One. 2010;5:e9085. 10.1371/JOURNAL.PONE.0009085. PMC2816710 20140211

[bib30] Liou AP, Paziuk M, Luevano JM et al. Conserved shifts in the gut microbiota due to gastric bypass reduce host weight and adiposity. Sci Transl Med. 2013;5:178ra41. 10.1126/SCITRANSLMED.3005687 PMC3652229 23536013

[bib31] Lloyd-Price J, Arze C, Ananthakrishnan AN et al. Multi-omics of the gut microbial ecosystem in inflammatory bowel diseases. Nature. 2019;569:655–62. 10.1038/s41586-019-1237-9. PMC6650278 31142855

[bib32] Lv BM, Quan Y, Zhang HY. Causal inference in microbiome medicine: principles and applications. Trends in Microbiology. 2021;29:736–46. 10.1016/J.TIM.2021.03.015. 33895062

[bib33] Mirzayi C, Renson A, Furlanello C et al. Reporting guidelines for human microbiome research: the STORMS checklist. Nat Med. 2021;27:1885–92. 10.1038/s41591-021-01552-x. PMC9105086 34789871

[bib34] Newgard CB, An J, Bain JR et al. A branched-chain amino acid-related metabolic signature that differentiates obese and lean humans and contributes to insulin resistance. Cell Metabolism. 2009;9:311–26. 10.1016/j.cmet.2009.02.002. PMC3640280 19356713

[bib35] Oka A, Sartor RB. Microbial-based and microbial-targeted therapies for inflammatory bowel diseases. Dig Dis Sci. 2020;65:757–88. 10.1007/S10620-020-06090-Z. PMC7311927 32006212

[bib36] Olamafar E, Yadegar A, Shahrokh S et al. Pathogenic potential of invasive mucosa-associated *Klebsiella pneumoniae* isolates in ulcerative colitis: characterization of virulence factors and inflammatory response. Immun Inflamm Dis. 2025;13:e70285. 10.1002/IID3.70285;ISSUE:ISSUE:DOI. PMC12521864 41090218

[bib37] Ou FS, Michiels S, Shyr Y et al. Biomarker discovery and validation: statistical considerations. J Thorac Oncol. 2021;16:537–45. 10.1016/J.JTHO.2021.01.1616. PMC8012218 33545385

[bib38] Pepe MS. The statistical evaluation of medical tests for classification and prediction. In: The Statistical Evaluation of Medical Tests for Classification and Prediction. 2023. 10.1093/OSO/9780198509844.001.0001 (30 October 2026. date last accessed).

[bib39] Rodriguez J, Hassani Z, Alves C et al. State of the art and the future of microbiome-based biomarkers: a multidisciplinary Delphi consensus. The Lancet Microbe. 2025;6:100948. 10.1016/j.lanmic.2024.07.011. 39243797

[bib40] Rothschild D, Weissbrod O, Barkan E et al. Environment dominates over host genetics in shaping human gut microbiota. Nature. 2018;555:210–5. 10.1038/nature25973. 29489753

[bib41] Ruan Z, Liu J, Zhao J. Causal associations between gut microbiota and type 2 diabetes mellitus subtypes: a mendelian randomization analysis. BMC Endocr Disord. 2025;25:79. 10.1186/S12902-025-01863-X PMC11931760 40122799

[bib42] Sayin SI, Wahlström A, Felin J et al. Gut microbiota regulates bile acid metabolism by reducing the levels of tauro-beta-muricholic acid, a naturally occurring FXR antagonist. Cell Metabolism. 2013;17:225–35. 10.1016/j.cmet.2013.01.003. 23395169

[bib43] Sinha R, Abu-Ali G, Vogtmann E et al. Assessment of variation in microbial community amplicon sequencing by the Microbiome Quality Control (MBQC) project consortium. Nat Biotechnol. 2017;35:1077–86. 10.1038/nbt.3981. PMC5839636 28967885

[bib44] Suzuki TA, Worobey M. Geographical variation of human gut microbial composition. Biol Lett. 2014;10:20131037. 10.1098/RSBL.2013.1037. PMC3949373 24522631

[bib45] Tremaroli V, Bäckhed F. Functional interactions between the gut microbiota and host metabolism. Nature. 2012;489:242–9. 10.1038/nature11552. 22972297

[bib46] Verster A, Petronella N, Green J et al. A Bayesian method for identifying associations between response variables and bacterial community composition. PLoS Comput Biol. 2022;18:e1010108. 10.1371/JOURNAL.PCBI.1010108. PMC9307184 35793382

[bib47] Vich Vila A, Collij V, Sanna S et al. Impact of commonly used drugs on the composition and metabolic function of the gut microbiota. Nat Commun. 2020;11:362. 10.1038/s41467-019-14177-z. PMC6969170 31953381

[bib48] Vrieze A, Van E, Holleman F et al. Transfer of intestinal microbiota from lean donors increases insulin sensitivity in individuals with metabolic syndrome. Gastroenterology. 2012;143:913–916.e7. 10.1053/J.GASTRO.2012.06.031. 22728514

[bib49] Walter J. Establishing microbiome causality to tackle malnutrition. Nat Microbiol. 2024;9:884–5. 10.1038/s41564-024-01653-6. 38503976

[bib50] Wang J, Qin J, Li Y et al. A metagenome-wide association study of gut microbiota in type 2 diabetes. Nature. 2012;490:55–60. 10.1038/nature11450. 23023125

[bib51] Wu H, Esteve E, Tremaroli V et al. Metformin alters the gut microbiome of individuals with treatment-naive type 2 diabetes, contributing to the therapeutic effects of the drug. Nat Med. 2017;23:850–8. 10.1038/nm.4345. 28530702

[bib52] Yatsunenko T, Rey FE, Manary MJ et al. Human gut microbiome viewed across age and geography. Nature. 2012;486:222–7. 10.1038/nature11053. PMC3376388 22699611

[bib53] Zeevi D, Korem T, Zmora N et al. Personalized nutrition by prediction of glycemic responses. Cell. 2015;163:1079–94. 10.1016/J.CELL.2015.11.001. 26590418

[bib54] Zhou C, Chen Z, Zhao L et al. A novel circulating miRNA-based signature for the early diagnosis and prognosis prediction of non–small-cell lung cancer. Clin Lab Anal. 2020;34. 10.1002/JCLA.23505 PMC7676218 33463758

[bib55] Zhou X, Zheng W, Kong W et al. Identification of circulating inflammation cytokines as a mediator of gut microbiota and type 2 diabetes mellitus: a Mendelian randomization study. Diabetol Metab Syndr. 2025;17:249. 10.1186/S13098-025-01792-8 PMC12220662 40604874

